# Exploring the Link Between Obstructive Sleep Apnea and Diabetes Mellitus: A Retrospective Single-Center Analysis

**DOI:** 10.3390/biomedicines13092261

**Published:** 2025-09-14

**Authors:** Mara Andreea Vultur, Dragoș Huțanu, Edith Simona Ianoși, Hédi-Katalin Sárközi, Corina Eugenia Budin, Maria Beatrice Ianoși, Mioara Szathmáry, Gabriela Jimborean

**Affiliations:** 1 Pulmonology Department, George Emil Palade University of Medicine, Pharmacy, Science, and Technology of Târgu Mureș, 540139 Târgu Mureș, Romania; mara.vultur@umfst.ro (M.A.V.); edith.ianosi@umfst.ro (E.S.I.); hedi.balog@umfst.ro (H.-K.S.); mioara.szathmary@umfst.ro (M.S.); gabriela.jimborean@umfst.ro (G.J.); 2 Doctoral School of Medicine and Pharmacy, George Emil Palade University of Medicine, Pharmacy, Science, and Technology of Târgu Mureș, 540139 Târgu Mureș, Romania; 3 Pulmonology Clinic, Mureș County Clinical Hospital, 540011 Târgu Mureș, Romania; ianosi.maria-beatrice@stud18.umfst.ro; 4 Pathophysiology Department, George Emil Palade University of Medicine, Pharmacy, Science, and Technology of Târgu Mureș, 540139 Târgu Mureș, Romania; corina.budin@umfst.ro

**Keywords:** obstructive sleep apnea (OSA), type 2 diabetes mellitus (T2DM), positive airway pressure (PAP) therapy

## Abstract

**Background**: Obstructive sleep apnea (OSA) is prevalent and often underdiagnosed, linking to cardiovascular disease, type 2 diabetes mellitus (T2DM), dyslipidemia, and cognitive decline. Coexistence with T2DM worsens patient outcomes. Positive airway pressure (PAP) therapy has demonstrated benefits in improving metabolic parameters and reducing comorbidities. **Methods**: This study examined the association between OSA and T2DM, focusing on therapy adherence. Overall, 73 patients from the pulmonology department, with diagnosed OSA and T2DM or prediabetes (PD) were compared to 72 OSA patients without diabetes. All underwent cardio-respiratory polygraphy. Data on demographics, comorbidities, and adherence were collected to evaluate disease severity and compliance. **Results**: Only 24% of patients were referred from cardiology or internal medicine. The STOP-BANG questionnaire accurately identified 83.4% of cases. Of the study group, 65.75% had T2DM, 34.2% had PD, and 16.5% received new diagnoses. T2DM patients had the highest BMI (40.19 kg/m^2^). Smoking prevalence exceeded European averages. These patients experienced more severe OSA and multiple comorbidities. PAP adherence increased from 73% to 86% after full financial coverage. **Conclusions**: Polygraphy remains an effective diagnostic tool. Patients with T2DM tend to have more severe OSA and comorbidities, underscoring the importance of early screening and increased awareness to improve management and outcomes.

## 1. Introduction

Obstructive sleep apnea (OSA) is a prevalent respiratory sleep disorder characterized by recurrent upper airway collapse during sleep, leading to significant medical and social consequences, including an increased risk of premature mortality [1]. Without appropriate treatment, OSA can result in severe complications due to recurrent nocturnal oxygen desaturation, heightened sympathetic tone, increased heart rate and hypertension, frequent arousals, poor sleep quality, daytime somnolence, and fatigue [2,3]. The associated complications are multifaceted and interrelated, including cardiovascular diseases (CVD) such as arterial hypertension, atrial fibrillation and other arrhythmias, ischemic cardiac and cerebrovascular diseases, as well as an elevated risk of type 2 diabetes mellitus (DM) and dyslipidemia (DYS). Furthermore, OSA has been implicated in cognitive decline, increased incidence of traffic accidents, diminished quality of life, and heightened mortality rates [2,4,5,6,7].

OSA has also been associated with the development and progression of type 2 DM. Patients with concurrent DM and OSA exhibit poorer glycemic control, increased insulin resistance, and a higher prevalence of diabetes-related complications [7,8,9,10,11]. Additionally, DM may exacerbate OSA through mechanisms such as neuropathy and myopathy. Treatment with positive airway pressure (PAP) has demonstrated therapeutic benefits in numerous studies; however, conflicting findings exist regarding its efficacy in improving glycemic control. Some studies have reported an improvement in DM management, with significant reductions in blood glucose levels and insulin resistance [12,13].

Despite these findings, inconsistencies remain due to the heterogeneity of study designs, the lack of standardized protocols regarding optimal continuous positive airway pressure (CPAP) settings, and variations in patient populations and concurrent antidiabetic treatments. Nonetheless, CPAP therapy has been shown to reduce certain OSA-related complications, lower glucose levels in patients with DM, and improve insulin sensitivity in individuals with prediabetes [14,15].

This study aims to elucidate the relationship between OSA and DM and assess the potential benefits of PAP therapy in improving metabolic and cardiovascular outcomes in affected individuals.

## 2. Materials and Methods

This study was conducted at the Pulmonology Clinic of Târgu Mureș County Hospital, Romania, as well as at the Pulmonology Department of the University of Medicine, Pharmacy, Science, and Technology “George Emil Palade,” Târgu Mureș, Romania. The study was conducted according to the guidelines of the Declaration of Helsinki, and approved by the Ethics Committee of the Mureș County Clinical Hospital, Târgu Mureș, Romania (decision number 9766/26 June 2025). The study center comprises five physicians with expertise in sleep medicine and one specialist in mechanical ventilation. The Pulmonology Clinic functions as a regional training center for pulmonology and sleep disorders. It operates as a public healthcare facility providing free medical care to insured patients. The sleep laboratory is equipped with three polygraphy devices, each with seven recording channels.

The study enrolled patients admitted to our center between October 2021 and July 2023. A total of 180 patients with clinical suspicion of sleep apnea were included based on their presenting signs and symptoms, medical history, scores on the Epworth Sleepiness Scale, results from the STOP-BANG questionnaire, and associated comorbidities all collected from existing medical records. All participants underwent cardio-respiratory polygraphy (PG) for diagnostic confirmation, followed by a second-night titration with positive airway pressure (PAP) therapy when indicated. The apnea-hypopnea index (AHI) was used as the primary measure for assessing the severity of obstructive sleep apnea syndrome (OSAS).

OSAS severity was classified using the AHI as follows: a value below 5 events per hour was considered normal; 5 to 15 events per hour indicated mild sleep apnea; 15 to 30 events per hour indicated moderate sleep apnea; and over 30 events per hour denoted severe sleep apnea [16]. Prediabetes was defined by fasting blood glucose levels ranging from 100 to 126 mg%. The parameters analyzed across both groups included gender and age distribution, correlations between age and AHI, relationships between body mass index (BMI) and AHI, and the presence of other comorbidities such as obesity, cardiovascular diseases, and dyslipidemia.

The inclusion criteria for our study were as follows: patients aged 18 years or older, admitted to the Pulmonology Clinic in a stable clinical condition; a diagnosis of obstructive sleep apnea (OSA) confirmed through cardio-respiratory polygraphy; and provision of informed consent along with a willingness to participate in the study.

Statistical analysis was realized with IBM SPSS Statistics version 26.0.0.0, where the distribution of quantitative data was tested through histograms, Q-Q plots, and finally through the Shapiro–Wilk test for normality, confirming the presence of non-parametrical data. Therefore, all results referring to quantitative data were expressed as the median (Q25–Q75). Qualitative data were analyzed using frequencies, with results expressed in n (%). Differences between the study groups were analyzed through the Mann–Whitney test for independent samples, Kruskal Wallis test, and Chi-square test accordingly, setting the significance limit at α = 0.05.

## 3. Results

A number of 145 patients with high suspicion of sleep disorders were found with a positive diagnosis of obstructive sleep apnea with a percentage of 80.55%, proving high value of the preprocedural clinical examination and the use of sleep questionnaires in the clinical practice.

The patients were subsequently categorized into two subgroups:

- Group 1 (n = 73): Patients with OSA and concurrent DM (n = 48) or prediabetes (PD) (n = 25).
- Group 2 (n = 72): Patients with OSA without DM or PD.

During the study period, the prevalence of diabetes mellitus (DM) and prediabetes (PD) was notably high among patients diagnosed with sleep apnea (SA), accounting for 73 out of 145 patients (50.3%). It is important to note that during 2021 and 2022, the diagnosis and hospitalization of SA and related conditions were significantly affected by the COVID-19 pandemic. Our clinic conducted a comprehensive review of patient data, documenting 17 patients in 2021 over three months, 61 patients in across whole 2022, and 67 patients in 2023 over eight months. The increase in patient numbers correlates with the relaxation of pandemic-related restrictions following COVID-19 vaccination efforts and an increased referral rate of patients with suspected apnea syndrome from colleagues across various specialties.

During the study period, patients were referred from various specialties, accounting for 55 out of 145 patients (37.93%). Specifically, referrals originated from the Pulmonology Clinic and ambulatory pulmonology services (62%), Internal Medicine Clinic (13.79%), Cardiology (10.34%), General Medicine (8.27%), Neurology Clinic (1.37%), and pre-surgical assessments performed by Intensive Care/Surgery teams prior to bariatric procedures (4.13%). These patients underwent polygraphy (PG) based on high clinical suspicion identified during examination and evaluation with the STOP-BANG questionnaire.

The Epworth Sleepiness Scale (ESS), used to assess diurnal somnolence (with a cutoff of over 10 points), was positive in 83 of 145 patients (57.24%). The STOP-BANG questionnaire (with a cutoff of over 5) showed a higher concordance with diagnosed sleep apnea cases, confirming 121 out of 145 patients (83.4%).

The vast majority of our patients presented with obstructive sleep apnea (OSA), accounting for 140 out of 145 patients (96.55%). Conversely, 5 patients (3.44%) exhibited central or mixed apnea. Polysomnography (PSG) was required in only four cases—two with complex central sleep apnea and two with neuromuscular conditions. Due to high costs, long waiting times, and delays in initiating treatment, PSG was accessible only in private clinics.

In our initial study cohort with sleep apnea (SA), 48 out of 73 patients (65.75%) had diabetes mellitus (DM), while 25 patients (34.2%) exhibited prediabetes (PD). The second group comprised 72 patients with SA who did not have DM. A notable finding was that 18 out of 73 cases (24.6%) were diagnosed with new-onset PD, and 6 cases (8.2%) with newly diagnosed DM, despite the presence of multiple comorbidities and symptoms. Therefore, the diagnosis of SA facilitated the detection of this significant disease in 32.87% of cases (24 out of 73). The median age of patients with DM and PD was 60 years, compared to 55 years in patients without DM.

We further analyzed the relationship between obesity, based on BMI, and the presence of DM and PD.

The non-diabetic patient group included a higher proportion of individuals with normal weight—9 out of 72 patients (12.5%)—compared to prediabetic patients, of whom 1 out of 25 (4%) were of normal weight, and none of the 48 diabetic patients (0%). Obesity remained a significant risk factor for sleep apnea (SA), with 112 out of 145 patients (77.2%) classified as obese and 23 patients (15.86%) as overweight. Among patients with DM, PD, and SA, the prevalence of obesity was notably high—61 out of 73 patients (83.56%)—compared to 70.83% in non-diabetic patients with *p* values achieveing the signification threshold of 0.05. The percentage of overweight individuals was similar across both groups, with 11 out of 73 patients (15.06%) in the diabetic/prediabetic group and 12 out of 72 patients (16.66%) in the non-diabetic group (Table 1).

The analysis revealed significant differences in body mass index (BMI) among the groups. The nondiabetic group had a mean BMI of 32.2 kg/m^2^, with a range of 20 to 47.83. The prediabetes group had a higher mean BMI of 36.4 kg/m^2^ (range: 27.76 to 43.45), while the diabetic group exhibited the highest mean BMI of 40.2 kg/m^2^ (range: 27.4 to 62). The differences among the groups were statistically significant (*p* < 0.01, with intergroup differences maintained as signifficant—*p* < 0.01 for all intergroup comparisons), indicating that higher BMI is associated with the progression from nondiabetes to prediabetes and diabetes (Table 2).

The distribution of obesity severity across the three groups showed no significant difference (*p* = 0.43). In the diabetes group, 22.7% had Grade I obesity, 20.5% had Grade II, and 56.8% had Grade III. The prediabetes group comprised 29.4% with Grade I, 29.4% with Grade II, and 41.2% with Grade III obesity. The nondiabetes group included 37.3% with Grade I, 23.5% with Grade II, and 39.2% with Grade III obesity (Table 3).

The comparison of gender distribution between the two groups demonstrated no statistically significant difference (*p* = 0.07). In the group with OSA and DM or prediabetes, 69.9% were males and 30.1% females. Conversely, in the group with OSA without DM or prediabetes, 55.6% were males and 44.4% females (Table 4a). Logistic regression analysis including sex as a predictor (Table 4b) showed that male patients had higher odds of belonging to the DM/prediabetes group compared to female patients (OR = 1.81, 95% CI 0.95–3.45, *p* = 0.07). This trend, although not statistically significant, is consistent with known sex-related differences in OSA and metabolic risk.

The distribution of age among patients with obstructive sleep apnea (OSA) differed significantly between those with and without diabetes or prediabetes (*p* = 0.01). Patients under 45 years of age were less likely to have DM or prediabetes, comprising only 4 of 73 (5.5%) in this group compared to 11 of 72 (15.3%) in patients without DM (*p* = 0.01). Conversely, the prevalence of DM or prediabetes increased among older patients, notably over 75 years, with 10 of 48 (20.8%) in the DM/prediabetes group versus 2 of 65 (3.1%) in the non-DM group, suggesting that age is a significant factor associated with metabolic comorbidities in OSA patients (Table 5).

Patients with OSA and diabetes or prediabetes were significantly older, with a mean age of 60.7 years compared to 51.1 years in those without diabetes (*p* < 0.01). The prevalence of smoking was higher in the DM/prediabetes group (52%) than in the non-diabetic group (38.8%), but this difference was not statistically significant (*p* = 0.11). Additionally, the BMI was significantly higher among patients with DM or prediabetes (median 38.3, IQR 27.4–62.1) compared to those without DM (median 32.2, IQR 20.4–47.83), with *p* = 0.04. Overall, older age and higher BMI are associated with the presence of DM or prediabetes in patients with OSA, whereas smoking prevalence did not differ significantly between groups (Table 6a). Logistic regression analysis (Table 6b) indicated that both age and BMI were significant predictors of DM/prediabetes in OSA patients. Each additional 10 years of age increased the odds of DM/prediabetes by more than threefold (OR = 3.25, 95% CI 1.55–6.82, *p* = 0.002), and each 5-unit increase in BMI doubled the odds (OR = 2.03, 95% CI 1.05–3.91, *p* = 0.037).

The analysis revealed significant differences in several cardiovascular and related conditions between patients with OSA and DM or prediabetes and those with OSA without DM. Patients with DM or prediabetes exhibited higher prevalence rates of hypertension (95% vs. 66.6%, *p* < 0.0001), ischemic cardiac disease (64.3% vs. 36.1%, *p* < 0.001), and a greater incidence of decreased professional productivity and early retirement (95% vs. 59.7%, *p* < 0.0001). Conversely, no significant differences were observed concerning peripheral arteriopathy, memory disorders, mood disposition, or dyslipidemia between the two groups (Table 7).

The average duration between the diagnosis of diabetes and the subsequent discovery of sleep apnea (SA) was notably prolonged, with a mean interval of 4 to 12 years. This delay contributes to the severity of both conditions and underscores the urgent need to initiate screening for SA shortly after a primary diagnosis of diabetes, particularly in patients with increased body mass index (BMI) or poorly controlled glycemic levels. Recognizing that obesity is a major risk factor for sleep apnea and can simultaneously exacerbate diabetes, early detection and intervention are essential to prevent disease progression and associated complications.

The comparison of sleep apnea severity between the two groups demonstrated significant differences. Patients with OSA and DM or prediabetes were more likely to have severe sleep apnea (78%) compared to those without DM (51.3%), with a *p*-value of 0.003. Conversely, mild sleep apnea was less common in the DM/prediabetes group (6.8%) than in the non-DM group (13.8%), while moderate sleep apnea was also less prevalent among patients with DM or prediabetes (15%) compared to those without DM (34.2%) (Table 8).

The management of sleep apnea (SA) during hospitalization and for home care involved a comprehensive and multidisciplinary approach. Patients received multiple sessions (3–5) of medical education during the initial days of hospitalization, conducted by the care team, including the attending physician and interns, to inform them about their condition and treatment options. They also received personalized diet plans or, when accessible, recommendations from specialized nutritionists to promote gradual weight loss. Patients were advised to gradually increase physical activity, such as walking over 5000 steps per day, using ergometric bicycles, or engaging in gym exercises involving multiple muscle groups; in some cases, pulmonary rehabilitation programs were recommended. During the second night of hospitalization, pressure titration with autoadjusting CPAP (APAP) devices was performed to determine the optimal positive airway pressure needed to eliminate nocturnal breathing events, facilitating effective home therapy.

CPAP remained the gold standard as first-line treatment, but for patients with low adherence, hypercapnia, overlap with COPD, or requiring high pressures, bilevel positive airway pressure (BiPAP) was recommended. A limited number of patients with mild SA received APAP devices, as they demonstrated better adherence. An interdisciplinary assessment was conducted to evaluate associated comorbidities, aiming to optimize overall health outcomes.

Post-discharge follow-up initially took place within 10 days to two weeks, focusing on clinical evolution, PAP compliance, residual apneas, oxygen desaturation below 90%, proper device technique, sleep quality, mask comfort, and air leaks. Further evaluations were scheduled at three months. One of the main barriers to treatment adherence was the lack of insurance coverage. During 2021–2022, many patients reported high device costs or rental fees as reasons for non-compliance. Additional reasons included fear of suffocation with the mask, device noise, and discomfort, leading to 31 out of 145 patients (21.37%) refusing to use PAP therapy at home.

In 2023, with full coverage introduced by the National Health Insurance, adherence notably improved, increasing from 70% to 88%, and daily device use rose from 73% to 86%.

The comparison between the two periods showed a significant increase in the number of patients using PAP device treatment and in treatment compliance. From 2021–2022 to February 2023, the proportion of patients on PAP therapy increased from 70.5% (55 patients) to 88% (59 patients), with a *p*-value of 0.014. Additionally, treatment adherence, measured by the number of days with PAP use over four hours, improved from 73% (57 patients) to 86% (58 patients), with a *p*-value of 0.04 (Table 9).

Over time, 8 patients of those who initially refused to use the device accepted the polygraphy reevaluation and titration in order to start PAP treatment.

The analysis revealed a significant difference in PAP therapy compliance between the two groups. Patients with OSA and DM or prediabetes had a lower rate of non-compliance (10.9%) compared to those without DM (31.9%), with a *p*-value of less than 0.001. Patients with DM and SA were more determined to follow the PAP treatment, explained by the increased severity of their disease (Table 10).

The data demonstrated significant improvements over time in several sleep and health parameters following APAP therapy. After the first night, 85.7% of patients experienced an improvement in sleep duration and quality, which increased to 98.4% at two weeks and reached 100% at three months. Diminution of daytime fatigue showed a similar trend, with 85.7% after one night and 100% at subsequent follow-ups. Additionally, Epworth sleepiness scores improved markedly, with 89.2% of patients showing reduction at two weeks and 95.4% at three months. The proportion of patients experiencing a decrease in nocturia was 38.5% at two weeks and 43% at three months. Control of blood pressure and absence of other cardiovascular events were observed in 87.7% and 92.3% of patients, respectively, at these time points. However, mask-related problems decreased significantly, from 32.75% after the first night to approximately 8–9% during follow-up (Table 11).

The results in patients without DM showed significant improvements following APAP therapy. After the first night, 83.3% experienced an enhancement in sleep duration and quality, increasing to 93.8% at two weeks and reaching 100% at six months (*p* = 0.01). Diminution of daytime fatigue was observed in 65% after one night, rising to 93.8% at two weeks, and achieving 100% at six months, with a *p*-value less than 0.01. Improvements in Epworth sleepiness scores were noted in 70% after the first night, escalating to approximately 96% at follow-up points (*p* < 0.001). No significant change was noted in nocturia rates over time (*p* = 0.80). Blood pressure control was achieved in 95.9% of patients at two weeks and remained stable at six months (*p* = 1.000). Mask-related problems showed a significant decrease from 20% after the first night to just 2% at six months (*p* < 0.001) (Table 12).

## 4. Discussion

The association between obstructive sleep apnea (OSA) and type 2 diabetes mellitus (T2DM) has been extensively documented. Nagayoshi et al. demonstrated in a cohort of 1453 patients with a follow-up of 12.8 years that the prevalence of diabetes is higher among OSA patients compared to those without OSA. Their findings indicated that OSA severity independently increases the risk of developing T2DM, regardless of obesity—supported by analyses from the Sleep Heart Health Study and the Atherosclerosis Risk in Communities Study [17].

Type 2 diabetes is characterized by abnormalities in glucose metabolism, resulting from defective insulin secretion or action. Diagnostic criteria include fasting blood glucose levels ≥ 7.0 mmol/L or 2-h postprandial glucose ≥ 11.1 mmol/L [18]. Several studies suggest that the impact of OSA on diabetes risk is comparable to other established factors such as obesity, genetic predisposition, sedentary lifestyle, and infections, and that this relationship is independent of obesity itself [19,20,21,22,23,24,25]. These findings underscore OSA as a significant risk factor for T2DM, emphasizing the importance of early detection and intervention to prevent or delay disease onset [21,22]. Obaseki et al. reported that 51.7% of diabetic patients with a BMI > 30 kg/m^2^ were at high risk for OSA, with significant associations observed between OSA risk, poor blood pressure control, and obesity [23].

The relationship between OSA and T2DM is bidirectional [24]. Diabetes can increase the risk of OSA through mechanisms such as peripheral neuropathy (leading to decreased muscle tone in the pharynx and larynx), diabetic myopathy, and central respiratory control disturbances [26,27,28,29]. Conversely, OSA exacerbates diabetes via mechanisms including nocturnal hypoxemia, systemic inflammation, insulin resistance, and increased sympathetic activity [2,3,5,7,9,10]. The coexistence of diabetes, obesity, and a sedentary lifestyle further accelerates disease progression and related complications [24,25]. Although obesity is a common risk factor for both conditions, OSA often remains underdiagnosed in diabetic or obese patients.

Habitual snoring, a hallmark symptom of OSA, has also been associated with disturbances in glucose metabolism, including elevated fasting glucose and HbA1c levels. In comparative studies, habitual snoring increased the risk for prediabetes (PD) by 1.84 times and T2DM by 2.24 times [30]. In our cohort, all patients with OSA were habitual snorers, and daytime sleepiness was associated with increased insulin resistance [22].

In our study, the average interval between diabetes diagnosis and sleep apnea assessment was 4 to 12 years—highlighting a significant delay, as sleep disorders can aggravate diabetes and its complications. This delayed diagnosis may explain the severity of sleep apnea observed. We recommend early investigation for OSA in diabetic patients, especially those with obesity or elevated BMI.

Our analysis identified advanced age, obesity (as measured by BMI), male gender, and diabetes-related complications as significant factors associated with severe OSA among T2DM and prediabetic patients. These findings align with a meta-analysis showing that the prevalence of OSA increases with age and BMI and is more common among males [31].

Early screening for OSA should be initiated soon after T2DM diagnosis, including questionnaires and objective studies such as polygraphy or polysomnography. Timely diagnosis allows for targeted treatment, which can improve both sleep apnea and diabetes outcomes. Several studies have demonstrated that positive airway pressure (PAP) therapy not only alleviates OSA symptoms but also improves glycemic control and reduces associated comorbidities [8,9,32,33]. In Indian populations, a high prevalence of OSA among diabetic patients—regardless of obesity—has been linked with poorer glycemic control and increased vascular complications.

CPAP therapy has been shown to significantly reduce HbA1c and fasting blood glucose levels, particularly in obese T2DM patients, with long-term adherence improving glucose metabolism and insulin sensitivity [12,13,28]. Adherence can be enhanced through patient education, behavioral interventions, and involvement of family or bed partners [32,33,34]. In our study, the use of oronasal masks and humidifiers resulted in 100% adherence at three months, supported by ongoing patient education and increased awareness among healthcare professionals across specialties. Additionally, since full financial coverage for PAP devices was introduced in Romania in February 2023, compliance rates have significantly improved. Given that sleep laboratories are primarily located within pulmonology clinics, referral pathways from other specialties are crucial for comprehensive sleep assessment and management.

## 5. Clinical Significance

The findings of this study have direct implications for clinical practice:

- Early recognition of OSA in diabetic and prediabetic patients enables timely intervention, reducing the risk of severe OSA and associated comorbidities.
- The STOP-BANG questionnaire proved highly accurate, supporting its use as a simple and efficient screening tool in primary care, cardiology, internal medicine, and endocrinology settings.
- Cardio-respiratory polygraphy represents a practical, cost-effective diagnostic method in settings where polysomnography is limited, making it suitable for broader implementation.
- PAP therapy showed clear benefits in terms of adherence, sleep quality, daytime functioning, and cardiovascular stability, particularly when financial barriers were removed.
- Incorporating routine OSA screening into diabetes care protocols may prevent disease progression, improve metabolic control, and ultimately enhance patient outcomes and quality of life.

## 6. Conclusions

- Patients with OSA and concurrent DM/prediabetes had significantly higher BMI, were older, and more frequently male compared to OSA patients without DM, highlighting the metabolic and demographic profile at risk.
- This group also showed a higher prevalence of cardiovascular comorbidities (hypertension, ischemic cardiac disease) and decreased professional productivity, emphasizing the clinical burden of the OSA–DM overlap.
- STOP-BANG demonstrated high accuracy as a screening tool, facilitating early identification of high-risk patients, while polygraphy proved to be an accessible and reliable diagnostic method in clinical practice.
- PAP therapy led to significant improvements in sleep quality, daytime fatigue, and cardiovascular stability; adherence rates increased substantially once full financial coverage was implemented, especially among patients with DM/prediabetes.
- The delayed recognition of OSA in diabetic patients (average delay of 4–12 years after DM diagnosis) underlines the importance of systematic screening soon after a DM or prediabetes diagnosis, particularly in individuals with obesity or poor glycemic control.
- Taken together, these findings stress the clinical necessity for early, integrated, and multidisciplinary management of patients with OSA and diabetes in order to reduce complications, improve quality of life, and optimize long-term outcomes.

## Figures and Tables

**Table 1:** Relation between nutritional status and the presence of DM or PD.

	OSA + DM/PD (n = 73)	OSA w/o DM/PD (n = 72)	*p* *
Body weight			
Normal weight	1 (1.36%)	9 (12.5%)	**0.02**
Overweight	11 (15.06%)	12 (16.66%)	
Obesity	61 (83.56%)	51 (70.83%)	

* Chi-square test.

**Table 2:** BMI average in patients groups.

	Nondiabetes (n = 72)	Prediabetes (n = 25)	Diabetes Melitus (n = 48)	*p* **
Body mass index (kg/m^2^)	32.2 (20–47.83)	36.4 (27.76–43.45)	40.19 (27.4–62)	**<0.01**

** Kruskal-Wallis test.

**Table 3:** Relation between obesity (considering BMI) and the presence of diabetes.

	Diabetes Group (n = 48)	Prediabetes Group (n = 25)	Nondiabetes (n = 72)	*p* *
Obesity	**n = 44**	**n = 17**	**n = 51**	0.43
Grade I	10 (22.72%)	5 (29.4%)	19 (37.25%)	
Grade II	9 (20.45%)	5 (29.4%)	12 (23.53%)	
Grade III	25 (56.8%)	7 (41.17%)	20 (39.2%)	

* Chi-square test.

**Table 4:** (**a**) Sex distribution and correlation with DM/PD of patients. (**b**) Logistic regression.

(**a**)								
		**Patients with OSA and DM or Prediabetes (n = 73)**		**Patients with OSA Without DM (n = 72)**			*p* *	
Males		51 (69.86%)		40 (55.55%)			0.07	
Females		22 (30.13%)		32 (44.44%)				
(**b**)								
	**β Coefficient**		**SE**		**OR**	**95% CI**		***p***
Male gender	0.59		0.33		1.81	1.55–6.82		0.07

(a) * Chi-square test.

**Table 5:** Age distribution.

	Patients with OSA and DM or Prediabetes (n = 73)	Patients with OSA Without DM (n = 72)	*p* *
Under 45 yo	4 (2.92%)	11 (15.27%)	**0.01**
45–60 yo	21 (28.76%)	27 (37.50%)	
61–75 yo	38 (52.05%)	32 (44.44%)	
Over 75 yo	10 (13.69%)	2 (2.77%)	

* Chi-square test.

**Table 6:** (**a**) General characteristics of patients. (**b**) Logistic regression.

(**a**)								
		**Patients with OSA and DM or Prediabetes (n = 73)**		**Patients with OSA Without DM (n = 72)**			***p***	
Age		60.7 (52.6–72.3)		51.1 (43.6–63.4)			<0.01 **	
Smoking		38 (52%)		28 (38.8%)			0.11 *	
BMI average		38.3 (27.40–62.10)		32.2 (20.40–47.83)			0.04 **	
(**b**)								
	**β Coefficient**		**SE**		**OR**	**95% CI**		***p***
Age (per 10 years)	1.18		0.38		3.25	1.55–6.82		**<0.01**
BMI (per 5 units)	0.71		0.35		2.03	1.05–3.91		**0.037**

(a) * Chi-square test; ** Mann-Whitney U test.

**Table 7:** Complication/comorbidities of sleep apnea in patients with and without DM.

	Patients with OSA and DM or Prediabetes (n = 73)	Patients with OSA Without DM (n = 72)	*p* *
Hypertension	70 (95%)	48 (66.6%)	**<0.0001**
Peripheral arteriopathy	5 (6.8%)	2 (2.7%)	0.25
Ischemic cardiac disease	47 (64.3%)	26 (36.1%)	**<0.001**
Memory disorders	34 (46.5%)	32 (44.4%)	0.79
Mood disposition	43 (58.1%)	41 (56.9%)	0.81
Dyslipidemia	45 (61.6%)	48 (66.6%)	0.52
Decrease in professional productivity and early retirement	70 (95%)	43 (59.7%)	**<0.0001**

* Chi-square test.

**Table 8:** Severity of sleep apnea.

	Patients with OSA and DM or Prediabetes (n = 73)	Patients with OSA Without DM (n = 72)	*p* *
Mild sleep apnea (AHI 5–15 events/hour)	5 (6.8%)	10 (13.8%)	**0.003**
Moderate sleep apnea (AHI 15–30 events/hour)	11 (15%)	25 (34.2%)	
Severe sleep apnea (AHI > 30 events/hour)	57 (78%)	37 (51.3%)	

* Chi-square test.

**Table 9:** Compliance to PAP treatment.

	2021 to February 2023 (n = 78)	February 2023–July 2023 (n = 67)	*p* *
Number of patients with PAP device treatment	55 (70.5%)	59 (88%)	**0.014**
Number of patients compliant to PAP therapy (>4 h/night)	57 (73%)	58 (86%)	**0.04**

* Chi-square test.

**Table 10:** Compliance to PAP treatment in our group of patients.

	Patients with OSA and DM or Prediabetes (n = 73)	Patients with OSA Without DM (n = 72)	p*
Failure to comply to PAP therapy	8 (10.9%)	23 (31.9%)	**<0.001**

* Chi-square test.

**Table 11:** Clinical impact of PAP therapy on patients with DM/PD.

	After the First Night Under APAP (n = 70)	After 2 Weeks (n = 65)	After 3 Months (n = 65)	*p* *
Improvement of the duration and quality of sleep	65 (85.7%)	64 (98.4%)	65 (100%)	0.09
Diminution of daytime fatigue	65 (85.7%)	65 (100%)	65 (100%)	0.07
Epworth score improvement (decrease with >2 points)	-	58 (89.23%)	62 (95.38%)	0.18
Decrease in nycturia	-	25 (38.46%)	28 (43%)	0.59
Control of blood pressure values and no other cardiovascular events	-	57 (87.7%)	60 (92.3%)	0.38
Mask problems	23 (32.75%)	5 (7.69%)	6 (9.23%)	**<0.0001**

* Chi-square test.

**Table 12:** Clinical impact of PAP therapy on the patients without DM/PD.

	After the First Night Under APAP (n = 60)	After 2 Weeks (n = 49)	After 6 Months (n = 49)	*p* *
Improvement of the duration and quality of sleep	50 (83.3%)	46 (93.8%)	49 (100%)	**0.01**
Diminution of daytime fatigue	39 (65%)	46 (93.8%)	49 (100%)	**<0.01**
Epworth score improvement	42 (70%)	47 (95.9%)	47 (95.9%)	**<0.001**
Decrease in nycturia	-	39 (79.6%)	38 (77.5%)	0.80
Control of blood pressure values and no other cardiovascular events	-	47 (95.9%)	47 (95.9%)	1.000
Masque problems	12 (20%)	2 (4%)	1 (2%)	**<0.001**

* Chi-square test.

## Data Availability

Dataset available on request from the authors.

## References

[1] Habumugisha J. (2025). Contemporary Approaches to Obstructive Sleep Apnea: A Review of Orthodontic and Non-Orthodontic Interventions in Children and Adults. Oral.

[2] Bonsignore M.R., Randerath W., Schiza S.E., Simonds A.K. (2023). ERS Handbook of Respiratory Sleep Medicine.

[3] Grunstein R.R., Stenlöf K., Hedner J., Sjöström L. (1995). Impact of obstructive sleep apnea and sleepiness on metabolic and cardiovascular risk factors in the Swedish Obese Subjects (SOS) study. Int. J. Obes. Relat. Metab. Disord..

[4] Jordan A.S., McSharry D.G., Malhotra A. (2014). Adult obstructive sleep apnoea. Lancet.

[5] Basoglu O.K., Tasbakan M.S. (2014). Elevated risk of sleepiness-related motor vehicle accidents in patients with obstructive sleep apnea syndrome: A case-control study. Traffic Inj. Prev..

[6] Tkacova R., Dorkova Z., Molcanyiova A., Radikova Z., Klimes I., Tkac I. (2008). Cardiovascular risk and insulin resistance in patients with obstructive sleep apnea. Med. Sci. Monit..

[7] Ip M.S., Lam B., Ng M.M., Lam W.K., Tsang K.W., Lam K.S. (2002). Obstructive sleep apnea is independently associated with insulin resistance. Am. J. Respir. Crit. Care Med..

[8] Fu Y., Xia Y., Yi H., Xu H., Guan J., Yin S. (2017). Meta-analysis of all-cause and cardiovascular mortality in obstructive sleep apnea with or without continuous positive airway pressure treatment. Sleep Breath..

[9] Saxena P., Singh D., Singh Y. (2022). Prevalence and impact of obstructive sleep apnea in type 2 diabetes mellitus: A descriptive cross-sectional study. Med. J. Armed Forces India.

[10] Botros N., Concato J., Mohsenin V., Selim B., Doctor K., Yaggi H.K. (2009). Obstructive sleep apnea as a risk factor for type 2 diabetes. Am. J. Med..

[11] Pamidi S., Aronsohn R.S., Tasali E. (2010). Obstructive sleep apnea: Role in the risk and severity of diabetes. Best Pract. Res. Clin. Endocrinol. Metab..

[12] Malik J.A., Masoodi S.R., Shoib S. (2017). Obstructive sleep apnea in Type 2 diabetes and impact of continuous positive airway pressure therapy on glycemic control. Indian J. Endocrinol. Metab..

[13] Zhao X., Zhang W., Xin S., Yu X., Zhang X. (2022). Effect of CPAP on blood glucose fluctuation in patients with type 2 diabetes mellitus and obstructive sleep apnea. Sleep Breath..

[14] Mokhlesi B., Grimaldi D., Beccuti G., Van Cauter E. (2017). Effect of one week of CPAP treatment of obstructive sleep apnoea on 24-hour profiles of glucose, insulin and counter-regulatory hormones in type 2 diabetes. Diabetes Obes. Metab..

[15] Pamidi S., Wroblewski K., Stepien M., Sharif-Sidi K., Kilkus J., Whitmore H., Tasali E. (2015). Eight hours of nightly continuous positive airway pressure treatment of obstructive sleep apnea improves glucose metabolism in patients with prediabetes. A randomized controlled trial. Am. J. Respir. Crit. Care Med..

[16] Soori R., Baikunje N., D’sA I., Bhushan N., Nagabhushana B., Hosmane G.B. (2022). Pitfalls of AHI system of severity grading in obstructive sleep apnoea. Sleep Sci..

[17] Nagayoshi M., Punjabi N.M., Selvin E., Pankow J.S., Shahar E., Iso H., Folsom A.R., Lutsey P.L. (2016). Obstructive sleep apnea and incident type 2 diabetes. Sleep Med..

[18] American Diabetic Association (2004). Diagnosis and classification of diabetes mellitus. Diabetes Care..

[19] Anothaisintawee T., Reutrakul S., Van Cauter E., Thakkinstian A. (2016). Sleep disturbances compared to traditional risk factors for diabetes development: Systematic review and meta-analysis. Sleep Med. Rev..

[20] Einhorn D., Stewart D.A., Erman M.K., Gordon N., Philis-Tsimikas A., Casal E. (2007). Prevalence of sleep apnea in a population of adults with type 2 diabetes mellitus. Endocr. Pract..

[21] Foster G.D., Sanders M.H., Millman R., Zammit G., Borradaile K.E., Newman A.B., Wadden T.A., Kelley D., Wing R.R., Sunyer F.X.P. (2009). Obstructive sleep apnea among obese patients with type 2 diabetes. Diabetes Care..

[22] Muraki I., Wada H., Tanigawa T. (2018). Sleep apnea and type 2 diabetes. J. Diabetes Investig..

[23] Obaseki D., Kolawole B., Gomerep S., Obaseki J., Abidoye I., Ikem R., Erhabor G. (2014). Prevalence and predictors of obstructive sleep apnea syndrome in a sample of patients with type 2 diabetes mellitus in Nigeria. Niger. Med. J..

[24] Aurora R.N., Punjabi N.M. (2013). Obstructive sleep apnoea and type 2 diabetes mellitus: A bidirectional association. Lancet Respir. Med..

[25] Umoh V.A., Akpan E.E., Ekrikpo U.E., Idung A.U., Ekpe E.E. (2020). The Risk of Obstructive Sleep Apnea among Patients with Type 2 Diabetes Mellitus. Niger. Med. J..

[26] Ficker J.H., Dertinger S., Siegfried W., König H., Pentz M., Sailer D., Katalinic A., Hahn E. (1998). Obstructive sleep apnoea and diabetes mellitus: The role of cardiovascular autonomic neuropathy. Eur. Respir. J..

[27] (2025). Association Pulmonaire du Quebec, Sleep Apnea. https://poumonquebec.ca/en/maladies/sleep-apnea.

[28] (2025). Johns Hopkins Medicine, the Dangers of Uncontrolled Sleep Apnea. https://www.hopkinsmedicine.org/health/wellness-and-prevention/the-dangers-of-uncontrolled-sleep-apnea.

[29] (2025). American Academy of Sleep Medicine, Patients with Type 2 Diabetes or Hypertension Must Be Evaluated for Sleep Apnea. https://aasm.org/patients-with-type-2-diabetes-or-hypertension-must-be-evaluated-for-sleep-apnea.

[30] Cho S.M.J., Lee H., Shim J.S., Kim H.C. (2020). Association of Snoring with Prediabetes and Type 2 Diabetes Mellitus: The Cardiovascular and Metabolic Diseases Etiology Research Center Cohort. Diabetes Metab. J..

[31] Andayeshgar B., Janatolmakan M., Soroush A., Azizi S.M., Khatony A. (2022). The prevalence of obstructive sleep apnea in patients with type 2 diabetes: A systematic review and meta-analysis. Sleep Sci. Pract..

[32] Punjabi N.M., Shahar E., Redline S., Gottlieb D.J., Givelber R., Resnick H.E. (2004). Sleep-disordered breathing, glucose intolerance, and insulin resistance: The Sleep Heart Health Study. Am. J. Epidemiol..

[33] Weaver T.E., Grunstein R.R. (2008). Adherence to continuous positive airway pressure therapy: The challenge to effective treatment. Proc. Am. Thorac. Soc..

[34] Budin C.E., Ciumarnean L., Maierean A., Rajnovean R., Gergely B.D., Man M., Aluas M., Cozma A., Bordea R.I. (2019). Therapeutic Alternatives with CPAP in Obstructive Sleep Apnea. J. Mind Med. Sci..

